# Antihypertensive Effects of Esaxerenone in Older Patients with Primary Aldosteronism

**DOI:** 10.1155/2023/6453933

**Published:** 2023-01-17

**Authors:** Masanori Fujimoto, Suzuka Watanabe, Katsushi Igarashi, Yutaro Ruike, Kazuki Ishiwata, Kumiko Naito, Akiko Ishida, Masaya Koshizaka, Sawako Suzuki, Yuki Shiko, Hisashi Koide, Koutaro Yokote

**Affiliations:** ^1^Department of Endocrinology, Hematology and Gerontology, Chiba University Graduate School of Medicine, Chiba 260-8670, Japan; ^2^Department of Diabetes, Endocrinology and Metabolism, Chiba University Hospital, Chiba 260-8670, Japan; ^3^Biostatistics Section, Clinical Research Center, Chiba University Hospital, Chiba 260-8670, Japan

## Abstract

**Design:**

Retrospective cohort study. *Patients*. The data was obtained from a total of 87 PA patients treated with esaxerenone. The treatment group comprised 33 patients who received esaxerenone as first-line therapy and 54 patients that switched from another MRA to esaxerenone. *Measurements*. Blood pressure (BP), plasma aldosterone concentration (PAC), plasma renin activity (PRA), serum potassium level, estimated glomerular filtration rate (eGFR), urinary albumin-creatinine ratio (UACR), and brain natriuretic peptide (BNP) were assessed before and after treatment with esaxerenone. Patients with overall reductions in their systolic or diastolic BP by 10 mmHg, or more, were considered responders. Unpaired *t*-tests of the biochemical and personal parameters between responders and nonresponders were run to find the most influencing characteristic for treatment success.

**Results:**

BP overall decreased after treatment with esaxerenone (systolic BP: *P*=0.025, diastolic BP: *P*=0.096). Serum potassium levels increased, while eGFR decreased (*P*=0.047 and 0.043, respectively). No patients needed a dose reduction or treatment discontinuation of esaxerenone based on the serum potassium and eGFR criteria. UACR and BNP decreased insignificantly. The responders were significantly older than the nonresponders of the esaxerenone treatment (*P*=0.0035).

**Conclusions:**

Esaxerenone was effective in older patients with primary aldosteronism.

## 1. Introduction

Primary aldosteronism (PA) accounts for 4–10% of hypertensive patients and is the most common cause of secondary hypertension [1, 2]. PA is caused by an autonomous aldosterone production from adrenal aldosterone-producing adenomas (APAs) or by bilateral adrenal hyperplasia defined as idiopathic hyperaldosteronism [3]. Patients with PA are more likely to have arrhythmias such as atrial fibrillation, cardiovascular disease, and stroke than patients with essential hypertension (EHT) matched for age and blood pressure [4, 5]. In a meta-analysis, PA patients had a significantly higher risk for developing stroke than those with essential hypertension, coronary artery disease, atrial fibrillation, and heart failure. PA is also associated with significantly higher risks of diabetes, metabolic syndrome, and left ventricular hypertrophy [5]. It is, therefore, important to accurately diagnose and treat patients with PA.

The pathogenesis of PA has not been fully elucidated yet, however many histological and genetical findings have been reported [6]. Multiple aldosterone-producing micronodules (APMs) (formerly known as aldosterone-producing cell clusters) appear to be a common histologic feature of bilateral PA [7]. Moreover, age-dependent accumulation of APMs in autopsy specimen of adrenal glands was reported, suggesting an age-dependent PA pathophysiology [8–10]. Also, aldosterone-producing driver-mutations are often found in APMs in normal adrenals, supporting that APMs acquiring somatic mutations induce an age-dependent aldosterone production [8, 10–12]. Thus, diagnosis and treatment of age-dependent aldosteronism has become more important.

The common treatment of PA is an adrenalectomy for patients with an APA, which reduces all-cause mortality better than mineralocorticoid receptor antagonist (MRA) treatment alone [13]. Additionally, an adrenalectomy for patients with APA reduced the risks of incident stroke, regression of left ventricular hypertrophy, and risk of atrial fibrillation [14–16]. However, MRAs are recommended for patients with bilateral PA and patients with PA unable or unwilling to undergo surgery and have shown to improve their hypertension. Furthermore, MRAs improve cardiac function and prognosis in patients with heart failure accompanied by reduced left ventricular ejection fraction [17–19].

Esaxerenone is an oral nonsteroidal MRA with higher MR-binding specificity than other MRA agents [20]. It has antihypertensive effects in patients with essential hypertension and PA and reduces microalbuminuria in patients with diabetic nephropathy [21–23]. Moreover, it is effective and well-tolerated in hypertensive patients with moderate kidney dysfunction [24]. However, the personal characteristics, such as age and sex, of patients that benefit most from esaxerenone treatment for PA have not been studied. Therefore, we investigated those characteristics of patients with PA under esaxerenone treatment to identify the patient type for whom this treatment is likely to be most effective.

## 2. Materials and Methods

### 2.1. Study Participants

This retrospective study enrolled 87 consecutive patients (mean age, 56.0 ± 11.9; females, *n* = 44) who had been diagnosed with PA and treated with esaxerenone at our institution, Chiba University Hospital, Japan, between May 2019 and July 2020. We obtained data before and on the next consultation day after treatment with esaxerenone (consultations mainly took place approximately 2 months after introduction, ranging from 1–3 months). The patients were diagnosed with PA based on the following criteria: (1) plasma aldosterone concentrations (PAC) of >120 pg/mL, determined via radioimmunoassay, (2) aldosterone-to-renin ratio (ARR) of >200, and (3) at least one positive confirmatory result for any of the following tests: captopril test (ARR >200 at 60 or 90 min after loading 50 mg of captopril); physiological saline challenge test (PAC >60 pg/mL 4 h after loading 0.9% saline); furosemide standing test (plasma renin activity (PRA) <2.0 ng/mL·h 2 hours after loading 40 mg of furosemide). All tests adhered to the Japan Endocrine Society guidelines [25]. The exclusion criteria were as follows: having other forms of secondary hypertension (e.g., renovascular hypertension, Cushing's syndrome, subclinical Cushing's syndrome, pheochromocytoma, or hypertension associated with a single kidney) or hypertensive crisis. The following measurements were taken before and after treatment with esaxerenone: (1) systolic and diastolic blood pressure (BP), (2) potassium level, (3) estimated glomerular filtration rate (eGFR), (4) B-type natriuretic peptide (BNP) level, and (5) urine albumin-to-creatinine ratio (UACR). Serum creatinine levels were used to calculate eGFRs, using the following equation: eGFR = 194 × serum creatinine − 1.904 × age − 0.287. A number of blood pressure stabilizers were permitted to be used during the study period: calcium channel blockers, angiotensin II receptor blockers, thiazides, beta-blockers, alpha-blockers, furosemide, renin inhibitors, and methyldopa. The PA patients included in this study received treatment with esaxerenone at varying doses, depending on new treatment group, dose of pretreatment MRB, and eGFR less than 60 ml/min/1.73 m^2^. They received either 1.25 mg/day, 2.5 mg/day, or 5 mg/day.

Since the observation period was defined as the period from the date of initiation of treatment to the date of continuation of esaxerenone without change of antihypertensive medication, the period of esaxerenone administration was the same as the follow-up period.

This study was approved by the ethics review board of the Chiba University Hospital, and it complied with the principles of the Helsinki Declaration.

### 2.2. Statistical Analyses

Data were analyzed with GraphPad Prism 7, version X (GraphPad Software, San Diego, CA, USA), and SAS version 9.4 (SAS Institute, Cary, NC, USA). Data are expressed as means ± standard deviation (SD), median, and interquartile range. The normality of the distribution was confirmed with Shapiro–Wilk's test. In case of normally distributed data, the two groups were compared using an unpaired or paired *t*-test. In case of nonnormally distributed data, the data were compared using Mann–Whitney *U* test or Wilcoxon test. Dose-stratified analysis were performed using ANOVA test. *P*-values of <0.05 denoted statistical significance. Subgroup analysis were performed for (1) concomitant group or noncomitant group of antihypertensive drugs and (2) ESA as the first therapy group and the switch from another MRA group.

## 3. Results

The average age of the patients was 56 ± 11.9 years (females, 50.6%). The median follow-up values were 9.1 weeks. Information regarding the subtype diagnosis for all patients was as follows: out of 87 cases, 53 cases are N.D. (not determined), 14 are unilateral, and 20 are bilateral. To stabilize their blood pressure, most patients were taking calcium channel blockers (66.7%). There were 33 patients in the treatment group with esaxerenone as first-line therapy, while 55 patients switched from another MRA to esaxerenone. Detailed information on the number of patients that took an antihypertensive agent and on the daily dose of esaxerenone can be found in Table 1.

Systolic BP and diastolic BP decreased after treatment with esaxerenone (*P*=0.025 and 0.096, respectively). UACR and BNP decreased; however, there were no significant reductions during the observation period. The serum potassium levels increased, while the eGFR decreased (*P*=0.047 and 0.043, respectively) (Table 2). Two patients (2.3%) had slightly elevated serum potassium levels of ≥5.0 mEq/L. No patient needed a dose reduction or the discontinuation of esaxerenone based on their serum potassium level and eGFR.

**Figure 1:**
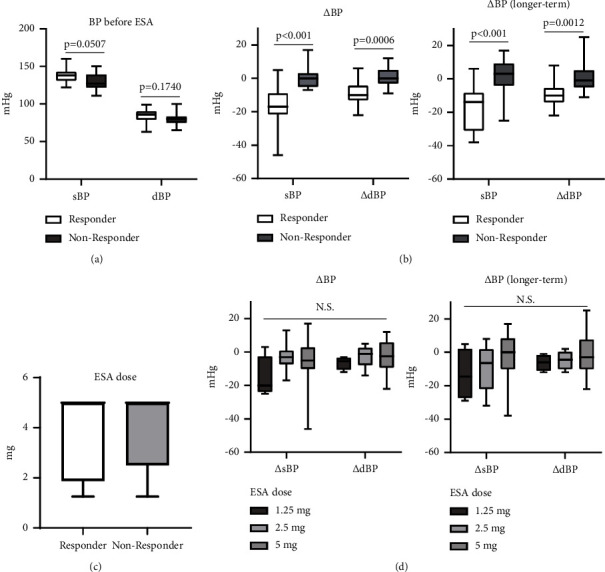
Blood pressure (BP) and doses of 13 responders and 19 nonresponders. (a) Systolic blood pressure (sBP) and diastolic blood pressure (dBP) before the introduction of esaxerenone (ESA) in each group (responder: white and nonresponder: grey bars). (b) BP changes after ESA introduction. Delta-BP (ΔBP) was calculated as the difference in sBP and dBP before and after treatment for each subject. (c) Dose of introduced ESA in each group. (d) Changes in sBP and dBP in each dose of esaxerenone. Each bar graph and error bar represent the mean ± SD.

**Figure 2:**
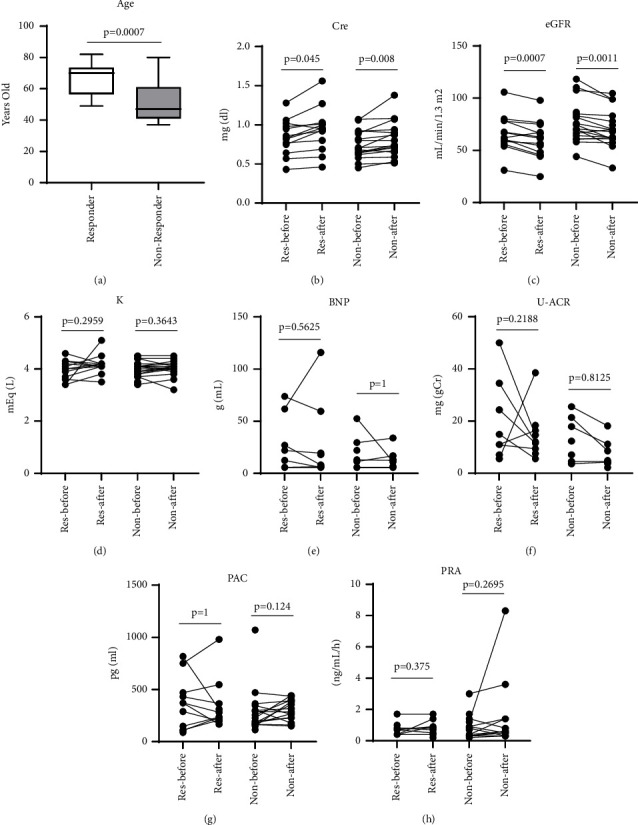
Comparison of clinical characteristics of 13 responders and 19 nonresponders. (a) Age. (b) Serum creatinine (Cre). (c) Estimated glomerular filtration rate (eGFR). (d) Serum potassium level (K). (e) Serum B-type natriuretic peptide (BNP) level. (f) Urine albumin-creatinine ratio (UACR). (g) Plasma aldosterone concentration (PAC). (h) Plasma renin activity (PRA). Bar graphs and error bars represent mean ± SD.

The blood pressure (BP) of 32 patients was measured before and after treatment, and those who had systolic or diastolic BP reductions of 10 mmHg, or more, were considered responders. Systolic BP before treatment was significantly higher in responders compared to nonresponders (*P*=0.025) (Figure 1(a)). Changes in systolic and diastolic, BP and ΔBP, after treatment were significantly greater in responders compared to nonresponders (*P*=0.00012 and *P*=0.00099, respectively) (Figure 1(b)). Similar changes were observed in longer-term treatment with esaxerenone (Figure 1(b) right). No significant differences between responders and nonresponders were observed with esaxerenone dose (Figure 1(c)). Changes in systolic and diastolic BP were studied in each dose of esaxerenone; however, the difference in dose-dependent effects was not significant (Figure 1(d)). The responders were significantly older than the nonresponders (*P*=0.0035) (Figure 2(a)). Biochemical parameters did not differ significantly between these two groups other than serum creatinine and estimated glomerular filtration rate (Figures 2(b)–2(h)). Clinical data of nonresponders and responders, and subtype diagnosis were shown in Table 3. Age was studied in the subgroups according to the presence or absence of antihypertensive drugs other than MRAs (Table 4). A subgroup analysis in patients who received esaxerenone as initial therapy and in patients who switched from other MRAs, and the results are shown in Table 5. Most of the parameters were similar to the overall observations with significant differences (*P* < 0.05).

## 4. Discussion

MRA is the recommended treatment for patients with PA who are unwilling to undergo surgery or who have contraindications for surgery [26]. In this study, we investigated the characteristics that maximize the effect of esaxerenone in hypertensive patients with PA, and assessed age, renal function, potassium levels, BNP, UACR, PAC, and PRA of the responders and nonresponders for esaxerenone. We found that the responders were significantly older than the nonresponders.

Conversely, the investigated biochemical parameters did not differ significantly between these two groups. Nevertheless, the observed changes of those measurements of PA patients after the treatment phase suggest an overall positive response. As such, both systolic and diastolic BP decreased in patients with PA after treatment with esaxerenone. Satoh et al. showed that significant reductions in systolic and diastolic BPs were observed from week 2 and continued through week 8 after treatment with esaxerenone for patients with PA [22]. A significant decrease was observed only in systolic BP in our study, most likely because approximately 60% of the cases had received pretreatment with other MRAs and were switched to a treatment with esaxerenone. Ito et al. reported that esaxerenone treatment showed persistent 24-hour antihypertensive effects and positive changes in diurnal BP in patients with essential hypertension [21]. We generally observed antihypertensive effects of esaxerenone in patients with PA, although the study period was limited and there were restrictions such as dose variation. The proportions of patients achieving the target sitting BP were 31.5% and 41.2% after being treated with esaxerenone 2.5 and 5 mg/day, respectively [21].

In our study, the serum potassium level significantly increased on average, and the effect of aldosterone was considered suppressed. Hyperkalemia with a serum potassium level of >5.0 mEq/L was observed in two cases; however, they were transient. Ito et al. reported that a serum potassium level of ≥5.5 mEq/L was observed in 12.1% of the patients with moderate kidney dysfunction receiving add-on treatment with esaxerenone. All increments in serum potassium levels were transient, and no patients met predefined serum potassium criteria for dose reduction or treatment discontinuation [24]. Furthermore, in the present analysis, the PRA increased to 1.0 ng/mL·h more on average. Previous reports indicated that PA patients with a PRA of <1.0 ng/mL·h had a higher incidence of cardiovascular events than those with a PRA of ≥1.0 ng/mL·h after surgery or medication, suggesting that the PRA of 1.0 ng/mL·h or higher in our study was desirable [27–30].

UACR and BNP values decreased in our study; however, there were no significant reductions. This result is not surprising. Adding esaxerenone to the existing renin-angiotensin system inhibitor therapy in patients with type 2 diabetes and microalbuminuria has shown to increase the likelihood of the normalization of the albumin levels and reduced progression of albuminuria [23]. In this study, eGFR decreased significantly, suggesting that the treatment with esaxerenone was effective for the patients with PA. Previously, BP, albuminuria, and eGFR decreased after 1 year of surgery or medical treatment with MRAs, and BP and albuminuria continued to decrease after 5.3 and 6.8 years, respectively, but eGFR remained stable without any further decrease [31].

While the biochemical parameters suggest the esaxerenone treatment is suitable as such for the chosen type of PA patients, the only prominent factor that determined an excellent response to the treatment was age. Older patients showed significantly better response than younger patients. Consistent with our findings, greater hypotensive effects were observed in older adults (>65 years old) by subgroup analyses on previous clinical trials of esaxerenone for the patients with EHT in the Japanese population [7]. However, although the precise mechanisms behind these findings are still unclear, several murine models on vascular aging have been studied. Krug et al., for example, reported an increased expression of the mineralocorticoid receptor (MR) in the aorta and enhanced sensitivity to aldosterone-mediated extracellular signal-regulated kinase 1/2 activation in aged rats. They also suggested that an increased MR signaling promoted age-associated inflammation, which in turn accompanies arterial aging [32, 33]. Interestingly, McCurley et al. showed that a deletion of smooth muscle cell-specific MR significantly decreased the systolic BP in aged mice (more than 7 months old) but not in young adult mice (4 to 7 months old) [32, 33]. Further studies are needed to address whether MR signaling is enhanced in blood vessels in aged humans and why the BP of older patients tends to decrease after a MR blockade. Excessive hypotension in older adults should be prevented. However, with careful observation, it is worth considering to lower BP or suppress the effects of aldosterone via a MR blockade in older patients.

We also studied blood pressure in longer-term treatment with esaxerenone (median follow-up: 13 months, mean: 14.7 months, S.D.: 10.2 months, 25% quartile: 4.8 months, 75% quartile: 23.3 months, nonresponders: 12.6 ± 9.3 months, responders: 15.2 ± 10.2 months). Nevertheless, SBP and DBP were significantly lower in responders than in nonresponders at 1–3 months. These results suggest that the high proportion of elderly patients in the responder group was not due to the short observation period. Also, according to clinical data of nonresponders and responders, and subtype diagnosis, there seemed to be no apparent difference in the proportion of subtype diagnoses between the two groups. In this cohort, there are many patients that switched from other MRAs, and some comparisons did not have significant differences, especially in the switching group, due to the smaller sample size. In contrast, the mean results show a trend similar to that seen in the whole group.

Retrospective studies are generally limited in the sense that the data have already been collected and the study design cannot be adjusted anymore in retrospective. Hence, we were limited by the relatively small sample size and short observational period. Finally, there was no washout period for other MRAs administered as pretreatment. Nonetheless, the strength of our study is that it led to candidate predictors of effectiveness regarding the treatment with esaxerenone in patients with PA.

## 5. Conclusions

We found that U-ACR and BNP decreased after treatment with esaxerenone. Further, esaxerenone was more effective in older patients with PA.

## Figures and Tables

**Table 1:** Clinical characteristics of PA patients (*n* = 87) that participated in the study. Values represent sample size (*n*) and percent (%), unless stated otherwise.

Patient characteristics	Total patients
Age (yr)	56.0^1^ ± 11.9^2^
Sex: females	44 (50.6)
Follow-up period (weeks, median)	9.1^1^ ± 4.4^2^
Diagnostic subtype	
Unilateral	14 (16.1)
Bilateral	20 (23.0)
N.D.^3^	53 (60.9)
Combination treatment with antihypertensive agents	
Calcium channel blockers	58 (66.7)
Angiotensin II receptor blockers	12 (13.8)
Thiazide	7 (8.0)
Beta blocker	6 (6.9)
Alpha blocker	4 (4.6)
Furosemide	3 (3.4)
Renin inhibitor	1 (1.1)
Methyldopa	1 (1.1)
Pretreatment with mineralocorticoid receptor agents	
Eplerenone	51 (58.6)
Spironolactone	3 (3.4)
None	33 (37.9)
Final dose of esaxerenone	
1.25 mg	7 (8.0)
2.5 mg	38 (43.7)
5 mg	42 (48.3)

^1^mean; ^2^standard deviation; ^3^not determined.

**Table 2:** Changes of blood pressure and biochemical parameters of PA patients before and after esaxerenone treatment. Data are mean ± SD.

	Before	After	*P*-value
Systolic blood pressure (mmHg)	134.2 ± 12.1	128.6 ± 12.5	0.0039^*∗*^
Diastolic blood pressure (mmHg)	81.1 ± 9.6	78.3 ± 8.5	0.0601
Plasma aldosterone concentration (pg/mL)	288.3 ± 211	321.5 ± 191	0.0125^*∗*^
Plasma renin activity (ng/mL·h)	0.86 ± 0.90	1.03 ± 1.51	0.2419
Potassium level (mmol/l)	3.94 ± 0.33	4.05 ± 0.41	0.1201
Creatinine (mg/dl)	0.82 ± 0.28	0.89 ± 0.28	<0.001^*∗*^
eGFR^1^ (mL/min/1.73 m^2^)	72.1 ± 20.7	66.5 ± 18.9	<0.001^*∗*^
UACR^2^ (mg/g creatinine)	130.4 ± 440	78.9 ± 300	0.131
BNP^3^ (pg/mL)	26.3 ± 38.8	25.3 ± 43.9	0.75

^1^eGFR: estimated glomerular filtration rate; ^2^UACR: urine albumin-to-creatinine ratio; ^3^BNP: B-type natriuretic peptide; ^*∗*^significant *P* value (*P* < 0.05).

**Table 3:** Statistics of non-responders and responders.

Non-responders									
		Mean	S.D.	Median	25%	75%	Minimum	Max	*P* value (before vs. after)
	Age	50.7	12.2	47	41	61	27	80	
	Sex: female	12 (63.2)							
	Laterality								
	ND	10							
	Unilateral	3							
	Bilateral	5							

	sBP	130.5	11.3	127	123.5	138.3	111	150	
	dBP	79.2	8.7	80	75	82.75	65	100	
	AftsBP	131.3	12.9	132	124	138	109	167	
	AftdBP	79.9	8.5	78	75	85.75	65	100	
	AftsBP.2	132.5789	16.65438	130	126.5	139.5	102	167	
	AftdBP.2	80.52632	8.421985	79	75	85.25	64	100	

Before	Cre before	0.7	0.2	0.71	0.64	0.82	0.45	1.07	0.008
	eGFR	77.1	19.4	72.4	64.7	85.1	44	118.3	0.0011
	K	4	0.3	4	3.8	4.1	3.4	4.5	0.3643
	BNP	14.1	14.4	5.8	5.8	12.2	5.8	52.6	1
	U-ACR	13.2	8.6	12.3	5.8	19.65	3.6	25.5	0.8125
	PAC	295.8	215.1	241	175.3	347	114	1070	0.124
	PRA	0.9	0.8	0.75	0.33	0.9	0.2	3	0.2695

After	Cre before after	0.8	0.2	0.74	0.68	0.89	0.51	1.38	
	eGFR	70.4	17.6	68.7	61.2	77.8	33	104.6	
	K	4	0.3	4.1	4	4.2	3.2	4.5	
	BNP	11.2	8.5	6.5	5.8	13.1	5.8	33.9	
	U-ACR	6.8	5.1	4.5	3.9	8.6	2.2	18.2	
	PAC	299.7	98.8	284	230	390.8	151	443	
	PRA	1.6	2.3	0.6	0.45	1.1	0.3	8.3	

Responders									

		Mean	S.D.	Median	25%	75%	Minimum	Max	*P* value (non-res vs. res)

	Age	61.9	16.8	70	56.5	73.5	49	82	0.0007
	Sex: female	6 (46.2)							
	Laterality								
	ND	7							
	Unilateral	2							
	Bilateral	4							

	sBP	138.6	10.6	138	131	140	122	160	0.0507
	dBP	84	10.7	86	81	90	63	99	0.174
	AftsBP	122.3	8.5	121	117	123	112	137	<0.0001
	AftdBP	75.6	5.9	75	72	77	68	89	0.0006
	AftsBP.2	120.1	11	118	115	128	100	137	<0.0001
	AftdBP.2	75.2	2.9	75	74	78	70	80	0.0028

	Cre before	0.8	0.2	0.83	0.75	0.98	0.43	1.28	0.0045
	eGFR	66.1	17.5	62.1	58.6	78	30.9	105.8	0.0007
	K	4	0.4	4	3.7	4.3	3.4	4.6	0.2959
	BNP	26.8	26.7	17.2	5.8	35.8	5.8	73.8	0.5625
	U-ACR	19.8	15.6	13	9.9	26.9	5.6	50	0.2188
	PAC	359.4	250.5	370	130.5	450.5	88	817	1
	PRA	0.8	0.4	0.7	0.4	0.8	0.4	1.7	0.375

	Cre before after	0.9	0.3	0.95	0.8	1.01	0.46	1.56	
	eGFR	60.1	18	61.4	48.2	67.2	24.9	97.9	
	K	4.2	0.4	4.1	4.1	4.2	3.5	5.1	
	BNP	26.4	35.4	12.95	5.975	19.375	5.8	115.9	
	U-ACR	14.9	9.7	12.1	9.4	16.4	5.6	38.5	
	PAC	352.4	246.6	257.5	213.3	351.3	167	981	
	PRA	0.8	0.5	0.8	0.5	0.9	0.2	1.7	

**Table 4:** Subgroup analysis; Concomitant of anti-hypertensive drugs.

Subgroup		Non-responders and responders		Total	*P*-value
		*N*	*R*		
Concomitant anti-hypertensive (−)		(*N* = 5)	(*N* = 4)	(*N* = 9)	
	Age				0.3758
	*N* (missing)	5 (0)	4 (0)	9 (0)	
	Mean (SD)	61.4 (17.49)	71.3 (12.45)	65.8 (15.43)	
	Median (IQR)	66.0 (47.0, 72.0)	75.5 (64.0, 78.5)	72.0 (53.0, 76.0)	
	Range	40.0, 82.0	53.0, 81.0	40.0, 82.0	

Concomitant anti-hypertensive (+)		(*N* = 14)	(*N* = 9)	(*N* = 23)	
	Age				0.00021
	*N* (missing)	14 (0)	9 (0)	23 (0)	
	Mean (SD)	49.9 (8.66)	67.4 (10.13)	56.7 (12.60)	
	Median (IQR)	49.0 (43.0, 54.0)	72.0 (61.0, 73.0)	54.0 (47.0, 67.0)	
	Range	39.0, 67.0	52.0, 84.0	39.0, 84.0	

**Table 5:** Subgroup analysis; ESA as first MRA or switched from another MRA.

Subgroup		Nonresponders and responders		Total	*P* value
		*N*	*R*		
ESA as first therapy		(*N* = 4)	(*N* = 4)	(*N* = 8)	
	sBP				0.0551
	*N* (missing)	4 (0)	4 (0)	8 (0)	
	Mean (SD)	124.0 (9.31)	141.3 (11.15)	132.6 (13.24)	
	Median (IQR)	126.0 (118.0, 130.0)	138.5 (134.0, 148.5)	132.0 (126.0, 138.5)	
	Range	111.0, 133.0	131.0, 157.0	111.0, 157.0	
	dBP				0.0144
	*N* (missing)	4 (0)	4 (0)	8 (0)	
	Mean (SD)	74.8 (2.06)	84.8 (5.50)	79.8 (6.58)	
	Median (IQR)	75.0 (73.5, 76.0)	86.0 (81.5, 88.0)	77.0 (75.0, 86.0)	
	Range	72.0, 77.0	77.0, 90.0	72.0, 90.0	
	ΔsBP				0.0041
	*N* (missing)	4 (0)	4 (0)	8 (0)	
	Mean (SD)	−1.3 (2.22)	−18.3 (7.23)	−9.8 (10.35)	
	Median (IQR)	−1.0 (−3.0, 0.5)	−20.0 (−22.5, −14.0)	−6.0 (−20.0, −1.0)	
	Range	−4.0, 1.0	−25.0, −8.0	−25.0, 1.0	
	ΔdBP				0.06
	*N* (missing)	4 (0)	4 (0)	8 (0)	
	Mean (SD)	−1.3 (5.32)	−9.3 (4.43)	−5.3 (6.23)	
	Median (IQR)	0.5 (−4.5, 2.0)	−9.0 (−13.0, −5.5)	−5.5 (−10.5, 0.5)	
	Range	−9.0, 3.0	−14.0, −5.0	−14.0, 3.0	
	ΔsBP1				0.2203
	*N* (missing)	4 (0)	4 (0)	8 (0)	
	Mean (SD)	−7.0 (12.36)	−17.8 (9.71)	−12.4 (11.78)	
	Median (IQR)	−3.0 (−14.5, 0.5)	−17.5 (−25.5, −10.0)	−10.0 (−23.5, −3.0)	
	Range	−25.0, 3.0	−29.0, −7.0	−29.0, 3.0	
	ΔdBP1				0.1146
	*N* (missing)	4 (0)	4 (0)	8 (0)	
	Mean (SD)	−2.5 (5.92)	−9.0 (3.83)	−5.8 (5.78)	
	Median (IQR)	−0.5 (−6.5, 1.5)	−10.0 (−12.0, −6.0)	−6.0 (−11.5, −0.5)	
	Range	−11.0, 2.0	−12.0, −4.0	−12.0, 2.0	
	ESAmg				0.024
	*N* (missing)	4 (0)	4 (0)	8 (0)	
	Mean (SD)	2.5 (0.00)	1.6 (0.63)	2.0 (0.65)	
	Median (IQR)	2.5 (2.5, 2.5)	1.3 (1.3, 1.9)	2.5 (1.3, 2.5)	
	Range	2.5, 2.5	1.3, 2.5	1.3, 2.5	

Switched from another MRA		(*N* = 15)	(*N* = 9)	(*N* = 24)	
	sBP				0.2855
	*N* (missing)	15 (0)	9 (0)	24 (0)	
	Mean (SD)	132.3 (11.43)	137.4 (10.84)	134.2 (11.27)	
	Median (IQR)	130.0 (122.0, 141.0)	138.0 (131.0, 140.0)	134.0 (125.0, 140.5)	
	Range	114.0, 150.0	122.0, 160.0	114.0, 160.0	
	dBP				0.4778
	*N* (missing)	15 (0)	9 (0)	24 (0)	
	Mean (SD)	80.4 (9.46)	83.7 (12.65)	81.6 (10.62)	
	Median (IQR)	81.0 (75.0, 83.0)	87.0 (81.0, 90.0)	82.5 (77.5, 88.5)	
	Range	65.0, 100.0	63.0, 99.0	63.0, 100.0	
	ΔsBP				0.0007
	*N* (missing)	15 (0)	9 (0)	24 (0)	
	Mean (SD)	1.3 (6.92)	−15.4 (14.08)	−5.0 (12.92)	
	Median (IQR)	2.0 (−5.0, 4.0)	−15.0 (−18.0, −10.0)	−5.0 (−10.0, 3.0)	
	Range	−7.0, 17.0	−46.0, 5.0	−46.0, 17.0	
	ΔdBP				0.0059
	*N* (missing)	15 (0)	9 (0)	24 (0)	
	Mean (SD)	1.2 (5.52)	−8.0 (9.37)	−2.3 (8.35)	
	Median (IQR)	0.0 (−3.0, 7.0)	−10.0 (−12.0, −4.0)	−2.5 (−6.0, 5.0)	
	Range	−5.0, 12.0	−22.0, 6.0	−22.0, 12.0	
	ΔsBP1				0.0001
	*N* (missing)	15 (0)	9 (0)	24 (0)	
	Mean (SD)	4.5 (8.60)	−18.9 (15.74)	−4.3 (16.27)	
	Median (IQR)	5.0 (0.0, 13.0)	−14.0 (−32.0, −10.0)	0.0 (−10.0, 7.0)	
	Range	−10.0, 17.0	−38.0, 6.0	−38.0, 17.0	
	ΔdBP1				0.0119
	*N* (missing)	15 (0)	9 (0)	24 (0)	
	Mean (SD)	2.3 (9.39)	−8.8 (9.97)	−1.8 (10.89)	
	Median (IQR)	−1.0 (−5.0, 8.0)	−10.0 (−16.0, −7.0)	−4.5 (−9.0, 5.5)	
	Range	−8.0, 25.0	−22.0, 8.0	−22.0, 25.0	
	ESAmg				0.5199
	*N* (missing)	15 (0)	9 (0)	24 (0)	
	Mean (SD)	4.4 (1.24)	4.7 (0.83)	4.5 (1.09)	
	Median (IQR)	5.0 (5.0, 5.0)	5.0 (5.0, 5.0)	5.0 (5.0, 5.0)	
	Range	1.3, 5.0	2.5, 5.0	1.3, 5.0	

## Data Availability

The data that support the findings of this study can be obtained from the corresponding author upon reasonable request.
